# Swimming against the flow—Environmental DNA can detect bull sharks (*Carcharhinus leucas*) across a dynamic deltaic interface

**DOI:** 10.1002/ece3.7101

**Published:** 2020-12-21

**Authors:** James Marcus Drymon, Katherine E. Schweiss, Emily A. Seubert, Ryan N. Lehman, Toby S. Daly‐Engel, Mariah Pfleger, Nicole M. Phillips

**Affiliations:** ^1^ Coastal Research and Extension Center Mississippi State University Biloxi MS USA; ^2^ Mississippi‐Alabama Sea Grant Consortium Ocean Springs MS USA; ^3^ School of Biological, Environmental, and Earth Sciences The University of Southern Mississippi Hattiesburg MS USA; ^4^ Department of Ocean Engineering and Marine Sciences Florida Institute of Technology Melbourne FL USA; ^5^ Oceana Washington DC USA

**Keywords:** eDNA, elasmobranch, estuary, habitat use, river

## Abstract

Human activities in coastal areas are accelerating ecosystem changes at an unprecedented pace, resulting in habitat loss, hydrological modifications, and predatory species declines. Understanding how these changes potentially cascade across marine and freshwater ecosystems requires knowing how mobile euryhaline species link these seemingly disparate systems. As upper trophic level predators, bull sharks (*Carcharhinus leucas*) play a crucial role in marine and freshwater ecosystem health. Telemetry studies in Mobile Bay, Alabama, suggest that bull sharks extensively use the northern portions of the bay, an estuarine–freshwater interface known as the Mobile‐Tensaw Delta. To assess whether bull sharks use freshwater habitats in this region, environmental DNA surveys were conducted during the dry summer and wet winter seasons in 2018. In each season, 5 × 1 L water samples were collected at each of 21 sites: five sites in Mobile Bay, six sites in the Mobile‐Tensaw Delta, and ten sites throughout the Mobile‐Tombigbee and Tensaw‐Alabama Rivers. Water samples were vacuum‐filtered, DNA extractions were performed on the particulate, and DNA extracts were analyzed with Droplet Digital™ Polymerase Chain Reaction using species‐specific primers and an internal probe to amplify a 237‐base pair fragment of the mitochondrial NADH dehydrogenase subunit 2 gene in bull sharks. One water sample collected during the summer in the Alabama River met the criteria for a positive detection, thereby confirming the presence of bull shark DNA. While preliminary, this finding suggests that bull sharks use less‐urbanized, riverine habitats up to 120 km upriver during Alabama's dry summer season.

## Graphical abstract

Estuarine, deltaic, and freshwater ecosystems are connected by mobile species that can increase ecosystem resilience by linking these otherwise disparate habitats. However, monitoring populations of mobile link species across multiple, expansive habitats presents logistical challenges. In this study, we use environmental DNA to provide the first evidence of bull shark habitat use 120 km upriver in the Alabama River. While preliminary, our findings provide a basis for further study and demonstrate the usefulness of this technique across similarly dynamic environments.

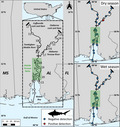

## 1 INTRODUCTION

Human alterations to the global landscape are accelerating shifts in ecosystem structure, function, and service at an unprecedented pace (Halpern et al., 2019). These trends are particularly evident in coastal areas marked by reductions in predatory species and losses of critical spawning and nursery habitats (Lotze et al., 2006). Increased urbanization of these coastal areas further contributes to changes in habitat by modifying hydrological processes and nutrient dynamics (Lee et al., 2006). Understanding how these anthropogenic activities cascade across ecosystems requires an understanding of how mobile species might act to link adjacent, but otherwise disparate, habitats (Lundberg & Moberg, 2003).

Mobile Bay, Alabama, is a dynamic, shallow, human‐impacted coastal ecosystem located in the north‐central Gulf of Mexico. Mobile Bay receives the fourth largest estuarine discharge in the continental United States (Dzwonkowski et al., 2011), 95% of which is accounted for by the Alabama and Tombigbee Rivers (Schroeder, 1978). The extensive discharge from these two rivers is also highly variable; average discharge during the wet season (late winter, early spring) is more than three times greater than average dry season discharge (late summer, early fall) (Webb & Marr, 2016). Ultimately, the nutrient‐rich discharge into Mobile Bay supports critical habitat, both for primary consumers like white shrimp (*Litopenaeus setiferus*, Linnaeus, 1767) and blue crab (*Callinectes sapidus*, Rathbun, 1896) (Rozas et al., 2013) and higher‐order consumers such as young‐of‐the‐year (YOY) bull sharks (*Carcharhinus leucas*, Müller and Henle, 1839) (Drymon et al., 2014).

Bull sharks are euryhaline generalists that often use freshwater environments as nursery areas (Grant et al., 2019) and thus may act as mobile links connecting the estuarine portions of Mobile Bay and freshwater reaches of the Alabama and Tombigbee Rivers. Acoustic tracking of YOY bull sharks demonstrates extensive use of the northern portion of Mobile Bay, an estuarine–freshwater interface known as the Mobile‐Tensaw Delta (MTD). Freshwater from the MTD enters Mobile Bay via two river systems. The Mobile‐Tombigbee river system discharges into the northwestern portion of Mobile Bay, along the industrial shores of the Port of Mobile. In contrast, the Tensaw‐Alabama river system discharges into the northeastern portion of Mobile Bay, an area with considerably less development (Ellis et al., 2011). Previous telemetry work suggests small‐scale habitat selection across these two adjacent areas. Acoustically tagged bull sharks were more frequently detected along the Tensaw‐Alabama portion of the MTD compared to the Mobile‐Tombigbee system (Drymon et al., 2014). This pattern suggests that YOY bull sharks may be linking freshwater and estuarine habitats in the MTD, but not equally across these two river systems. Determining how YOY bull sharks connect these habitats is critical given the role of mobile links in ecosystem resilience (Lundberg & Moberg, 2003), yet cost‐prohibitive when using traditional techniques such as fisheries‐independent monitoring or passive acoustic telemetry. Therefore, the objective of the current study was to use a newly developed bull shark environmental DNA (eDNA) assay (see Schweiss et al., 2020) to examine the potential for eDNA approaches to detect bull sharks across this highly dynamic deltaic interface.

## 2 METHODS

**Figure 1:**
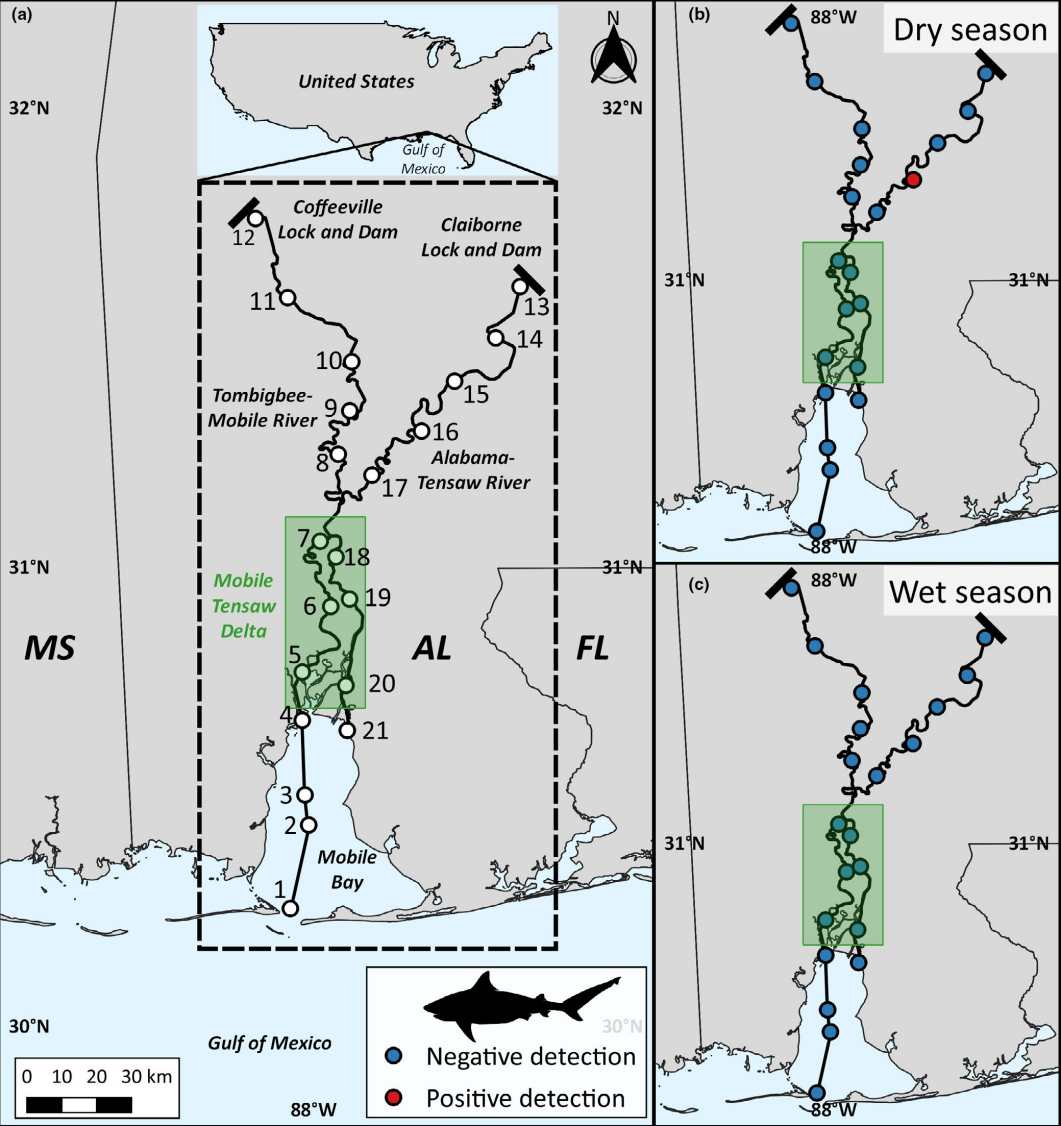
(a) Study area of environmental DNA surveys for bull sharks in the Mobile‐Tombigbee and the Tensaw‐Alabama Rivers, including the Mobile‐Tensaw Delta (green box). (b) Dry season (August 2018) and (c) wet season (February 2018) water collection sites are indicated with circles; blue are negative detections and red are positive detections

Water samples were collected at a series of estuarine, deltaic, and freshwater sites in Alabama once in the wet winter season (February 19–20, 2018) and once in the dry summer season (August 21–22, 2018). In total, five estuarine sites were sampled from Dauphin Island to the northern extent of Mobile Bay, six sites were sampled within the MTD, and ten freshwater sites were sampled in two river systems: the Mobile‐Tombigbee and the Tensaw‐Alabama. In each river system, sites spanned ~190 km north of the mouth to just south of the Coffeeville Lock and Dam and Claiborne Lock and Dam in the Tombigbee and Alabama Rivers, respectively (Figure 1a). All sampling sites were spaced 15–25 km apart (Figure 1a). At each site, 5 × 1 L water samples and environmental data, including temperature (°C), salinity (psu), dissolved oxygen (mg/L), and depth (m), were collected approximately 0.5 m below the surface of the water. While bull sharks are known to use the entire water column, previous studies have identified surface water sampling as the most suitable method for eDNA collection in headwaters (Katano et al., 2017). In Mobile Bay, water samples were collected in proximity to structures (e.g., oil rig and lighthouse), and at all riverine sites, samples were collected across the width of the river.

All eDNA field and laboratory protocols and controls followed Schweiss et al. (2020), and new gloves were used at each sampling site. Water samples were collected in the field using sterile, 1 L high‐density polyethylene Nalgene^®^ bottles and stored on ice in clean marine coolers, or frozen, until water filtration (see Schweiss et al., 2020). In addition to the filtration, DNA extraction, and PCR‐negative controls described in Schweiss et al. (2020), negative collection controls were also included. The negative collection controls consisted of autoclaved deionized water, which was taken onto the boat and placed in a clean marine cooler on ice with field samples to test for field contamination (e.g., Jerde et al., 2011). All negative control samples (collection, filtration, DNA extraction, and PCR) were processed and analyzed in replicates of five, according to the protocols of Schweiss et al. (2020), and were defined as negative if they did not meet any of the criteria for positive detections. Water samples were vacuum‐filtered in a laboratory using 47‐mm‐diameter, 0.8*‐*μm nylon filters (Cole Parmer^®^) and preserved in 95% ethanol at room temperature (see Schweiss et al., 2020).

Total eDNA was extracted from ¼ of each filter following the Goldberg et al. (2016) QIAGEN^®^ DNeasy^®^ Blood & Tissue Kit protocol incorporating the QIAshredder™ spin columns. A species‐specific bull shark Droplet Digital™ PCR (ddPCR™) assay was used to target a 237‐base pair fragment of the mitochondrial NADH dehydrogenase subunit 2 (mtDNA ND2) gene using the reaction mixtures and ddPCR™ cycling conditions described in Schweiss et al. (2020). Five replicates (5% of the total eDNA extract) were run for each sample on the Bio‐Rad^®^ QX200™ AutoDG™ Droplet Digital™ PCR System (Droplet Generator instrument no. 773BR1456, Droplet Reader instrument no. 771BR2544) platform. Positive detections were defined as samples with at least one ddPCR™ replicate that met all three analysis criteria: (a) Droplets were above the manual threshold of 3,000 amplitude, (b) droplets were within the known positive droplet range for the target species (e.g., 4,500–6,000 amplitude), and (c) the concentration (copies/μl) was greater than or equal to the refined Limit of Detection (LoD) of 0.09 copies/μl for the assay (https://doi.org/10.5061/dryad.m0cfxpp29), using the Rare Event Detection (RED) analysis in Bio‐Rad® QuantaSoft™ software.

## 3 RESULTS

One water sample, collected from the Alabama River (site 16) at 11:00 a.m. on August 22, 2018, met all three criteria for a positive detection (0.10 copies/µl), indicating the presence of bull shark DNA ~ 120 km upriver in the dry summer season (Figure 1b). Freshwater discharge during the time of sampling was 510 m^3^/s,^1^ characteristic of dry season flow conditions. At this site, the water was warm (29.6°C), normoxic (7.5 mg/L), and fresh (0.07 psu) (Table 1). Water samples collected at all other sites during the dry summer season and wet winter season did not meet any of the criteria for positive detections. None of the collection, filtration, DNA extraction, and PCR controls met any of the three analysis criteria for positive detections (see Schweiss et al., 2020); therefore, samples were considered free from contamination by target DNA.

**Table 1:** Environmental parameters collected at each location during wet (February 2018) and dry (August 2018) seasons

Station	Latitude	Longitude	Depth (m)	Temperature (°C)		Salinity (psu)		DO (mg/L)	
				Wet	Dry	Wet	Dry	Wet	Dry
1	30.2560	−88.0510	4.7	14.0	28.5	2.61	23.86	9.66	6.04
2	30.4380	−88.0110	5.2	14.8	28.1	1.28	14.84	9.89	6.52
3	30.5380	−87.9970	5.6	13.1	27.6	0.33	13.00	9.50	7.15
4	30.6660	−88.0250	1.6	11.8	28.6	0.74	4.17	9.44	6.65
5	30.7710	−88.0250	1.4	12.1	30.0	0.08	1.49	9.23	6.75
6	30.9140	−87.9630	5.1	11.6	30.3	0.07	0.08	9.09	6.94
7	31.0560	−87.9860	4.6	11.5	29.7	0.07	0.09	9.20	6.87
8	31.2460	−87.9467	4.8	11.7	29.5	0.07	0.10	9.33	6.84
9	31.3400	−87.9215	8.2	11.3	29.0	0.06	0.10	9.38	6.92
10	31.4470	−87.9172	5.9	11.5	30.0	0.06	0.12	9.23	7.65
11	31.5870	−88.0569	5.4	11.5	30.4	0.06	0.12	9.32	8.08
12	31.7570	−88.1290	4.3	11.4	30.7	0.06	0.12	9.27	7.82
13	31.6110	−87.5505	4.9	11.5	29.2	0.06	0.07	10.67	8.50
14	31.4990	−87.5505	7.5	11.7	29.1	0.06	0.07	10.50	7.81
15	31.4050	−87.6931	2.8	11.7	29.7	0.07	0.07	10.62	7.56
16	31.2960	−87.7651	5.0	12.4	29.4	0.07	0.07	9.97	7.50
17	31.2000	−87.8731	5.0	12.1	29.8	0.06	0.07	9.67	6.87
18	31.0270	−87.9560	5.0	12.4	29.2	0.07	0.08	9.00	6.50
19	30.9300	−87.9220	1.7	13.7	31.1	0.07	0.09	8.98	7.88
20	30.7340	−87.9340	6.2	13.2	30.2	0.07	0.12	9.24	7.06
21	30.6440	−87.9270	5.1	13.1	30.5	0.07	0.20	9.20	7.56

## 4 DISCUSSION

The headwaters that pass through the MTD and feed the Mobile Bay estuary encompass the richest freshwater fauna in North America (Boschung & Mayden, 2004; Lydeard & Mayden, 1995), including many rare and endemic species. Monitoring the populations of such biodiverse fish fauna across such a vast expanse can be challenging. Our findings provide evidence that bull sharks can occupy freshwater upstream habitat in the Alabama River and further demonstrate the ability of eDNA to identify rare species in Alabama rivers (e.g., Alabama Sturgeon *Scaphirhynchus suttkusi*, Pfleger et al., 2016). To place our findings into context, a synthesis of 150 years of survey data collected from 3,716 sampling locations throughout Alabama's many rivers and the Mobile Basin noted only two bull sharks, both of which were located in estuarine waters south of the MTD (Mettee et al., 1996). Thus, our findings represent the first scientific evidence of bull shark habitat use in this freshwater riverine system.

Although our data are limited to one survey in each of the dry summer and wet winter seasons, they provide preliminary information on potential spatial and temporal patterns of the occurrence of bull sharks in this region. The positive eDNA sample from site 16 suggests that at least one bull shark was present at that site or further upstream. The Alabama River contributes to one of the largest discharge volumes in the continental United States (Dzwonkowski et al., 2011); therefore, shed eDNA is expected to disperse downriver from the source relatively quickly (e.g., Jane et al., 2014; Wacker et al., 2019). While dispersing, eDNA molecules are subject to biological and physical degradation, which is accelerated in fresh, warm (e.g., >20°C) waters with high levels of microbial activity (Collins et al., 2018; Strickler et al., 2015). The positive bull shark eDNA detection in this study occurred during the dry summer season, when water temperatures were warm and discharge in the Alabama River was relatively low (i.e., less than ~500 m^3^/s, Webb & Marr, 2016). Given these conditions, the persistence time of the detected bull shark DNA was likely short. Studies of DNA degradation under similar conditions found an eDNA half‐life of ~3 hr, with a life span of ~6 hr for bony fish (Tsuji et al., 2017). When combined with a lack of positive detections north of this site, this suggests that bull sharks were likely present within the vicinity of the positive detection or slightly north.

Several lines of evidence indicate that the positive bull shark detection at site 16 was most likely a YOY individual. Long‐term gillnet sampling demonstrates that the shark assemblage in Mobile Bay is dominated by bull sharks, approximately 80% of which are YOY (Bethea et al., 2015). Similar size‐based segregation has been widely demonstrated for bull sharks off the east (Curtis et al., 2013) and west (Simpfendorfer et al., 2005) coasts of Florida and in Texas estuaries (Matich et al., 2020), where YOY individuals preferentially occupy riverine habitats. In Florida, YOY bull sharks move upriver into shallow freshwater habitats during periods of low discharge to take advantage of pulsed resources (Matich & Heithaus, 2014) while residing in a low‐mortality environment (Heupel & Simpfendorfer, 2011). The positive bull shark detection from this study occurred in freshwater habitat ~120 km upriver during the dry season; thus, freshwater refugia in Alabama may provide benefits similar to the riverine habitat occupied by conspecifics in Florida and Texas.

There were no positive detections in the highly urbanized Mobile‐Tombigbee system, nor at any sites in either river system during the wet winter season. The only positive eDNA bull shark detection in the present study was in the less‐urbanized Tensaw‐Alabama system, corroborating the findings of Drymon et al. (2014) that bull sharks have an affinity for less‐urbanized habitats in the northeastern portion of Mobile Bay. Bull sharks also have an affinity for warmer (i.e., >20°C) waters, which could account for the apparent seasonal presence of bull sharks in the freshwater habitats, evidenced by a lack of positive detections during winter sampling. The average temperature across sites during the wet winter season was 12.3°C (*SE* = 0.22), well below the affinity range of bull sharks. However, interpretation of negative detections requires careful consideration of the potential for false negatives as a result of sampling errors. Modifications to the methods, such as collecting water samples from bottom waters, extracting DNA from a larger portion of each filter, screening more of each DNA extract for target DNA, or targeting a second locus could reveal additional positive detections.

While our preliminary findings show clear promise, additional surveys are needed to understand the spatial and temporal extent of riverine habitat use by bull sharks in Alabama. Additional eDNA surveys are needed in each river to assess whether the preliminary spatial and temporal patterns described here are evident in replicate surveys. These efforts would be most efficient when coupled with high‐resolution hydrographic models (e.g., Webb & Marr, 2016) that could attempt to characterize eDNA particle residence and flushing, allowing for estimations of the origins of DNA sources. Such exploratory “pilot” eDNA surveys are useful for identifying appropriate seasons and general locations where more targeted, multi‐gear sampling can take place. Survey designs that make use of relatively low‐cost and complementary pilot eDNA surveys, which are free from the caveats of more traditional sampling gears such as gillnets, may have a higher likelihood of successful captures. This approach is especially important when targeting rare species, thereby expanding the use of limited resources.

Increasing urbanization of coastal regions and hydrological modifications to riverine ecosystems is accentuating the burden placed on species that use these habitats (Grant et al., 2019). This is particularly acute for YOY bull sharks in Alabama's riverine system, an ecosystem referred to as “North America's neglected hotspot” (Lydeard & Mayden, 1995). While preliminary, our findings add to a body of literature documenting the importance of freshwater habitats to bull sharks in both the eastern (Simpfendorfer et al., 2005) and western (Matich et al., 2020) Gulf of Mexico. By functioning as predatory mobile links across marine and freshwater habitats in coastal Alabama, bull sharks play a critical role in this ecosystem through their ability to influence prey abundance and behavior, maintain biodiversity, and buffer against invasive species (see Ferretti et al., 2010; Ritchie et al., 2012). In the north‐central Gulf of Mexico, eDNA represents a powerful tool to identify how future changes in freshwater discharge and/or urbanization may impact habitat use by bull sharks, with important implications for the overall health of this system.

## CONFLICT OF INTEREST

None declared.

## AUTHOR CONTRIBUTIONS

**J. Marcus Drymon:** Conceptualization (lead); funding acquisition (lead); investigation (lead); supervision (lead); writing‐original draft (lead); writing‐review & editing (lead). **Katherine E. Schweiss:** Investigation (supporting); writing‐review & editing (supporting). **Emily A. Seubert:** Investigation (supporting); writing‐review & editing (supporting). **Ryan N. Lehman:** Investigation (supporting); writing‐review & editing (supporting). **Toby S. Daly‐Engel:** Conceptualization (supporting); writing‐review & editing (supporting). **Mariah Pfleger:** Conceptualization (supporting); writing‐review & editing (supporting). **Nicole M. Phillips:** Conceptualization (lead); funding acquisition (lead); investigation (lead); supervision (lead); writing‐original draft (lead); writing‐review & editing (lead).

## Data Availability

All data associated with this publication (sample site coordinates, environmental data, number of copies of target DNA/ul for each sample and limit of detection plot) can be accessed on Dryad (https://doi.org/10.5061/dryad.m0cfxpp29).

## ENDNOTE

^1^ waterdata.usgs.gov.
